# Assessment of Malar Prominence in Adolescents: Evaluating the Diagnostic Accuracy of a New Angle for Vector Profile Classification

**DOI:** 10.7759/cureus.79289

**Published:** 2025-02-19

**Authors:** Aishwarya Sonawane, Aameer Parkar, Chetan Patil, Snehal V Bhalerao, Pradeep Kumar, Priyanka Razdan

**Affiliations:** 1 Department of Orthodontics and Dentofacial Orthopedics, Yogita Dental College and Hospital, Khed, IND; 2 Department of Pediatric and Preventive Dentistry, Yogita Dental College and Hospital, Khed, IND

**Keywords:** angle, cephalometric, diagnosis, malar bone, relationship, vector

## Abstract

Introduction: The prominence of the malar region plays a crucial role in facial aesthetics; however, standardized diagnostic parameters for assessing midfacial deficiencies remain limited. This study introduced and evaluated a novel cephalometric parameter, the double W-key ridge (DWK) angle (formed between the double W plane and the key ridge point), in comparison with the established sella-nasion-orbitale (SNO) angle for assessing malar prominence. This study aimed to present a novel perspective for evaluating malar prominence, referred to as the DWK angle. The objectives of this study were to compare the mean SNO and DWK angles between positive and negative vector profiles, assess their correlation, evaluate their diagnostic accuracy using receiver operating characteristic (ROC) analysis, and examine the influence of sex on these parameters.

Materials and methods: This retrospective study analyzed the lateral cephalograms and profile photographs of 60 young adolescents (aged 11-14 years) from the Department of Orthodontics, Yogita Dental College. Based on clinical photographs, records were classified into positive and negative vector profile groups (n=30 each). The same observer obtained cephalometric measurements of the SNO and DWK angles. Reliability testing was conducted using the intraclass correlation coefficient. Statistical analyses, including Mann-Whitney U tests, Spearman’s rank correlation, mixed-model analysis, and ROC curve analysis using the area under the curve (AUC), were performed to assess the relationship, diagnostic accuracy, and potential sex differences in these measurements.

Results: Mean SNO and DWK angles were significantly higher in the positive vector group (SNO, 53.97°; DWK, 104.2°) than in the negative vector group (SNO, 44.1°; DWK, 94.6°) (p<0.001). A strong positive correlation (r=0.74, p=0.001) was observed between these two angles. ROC analysis demonstrated high diagnostic accuracy for both angles (AUC: 0.947 for SNO and 0.961 for DWK), with a sensitivity and specificity of 90%. No significant sex-based differences were found in either of the vector groups.

Conclusion: DWK angle is a stable and reliable cephalometric parameter for differentiating between adolescents' positive and negative vector profiles. The strong correlation and high diagnostic accuracy suggest that both SNO and DWK angles can effectively be utilized in orthodontics and maxillofacial planning to assess malar prominence.

## Introduction

The attainment of optimal integration of facial aesthetics alongside properly aligned occlusion remains a principal aim within the domain of orthodontic intervention. The significance of conducting a comprehensive facial assessment is ensuring that any orthodontic or orthopedic modifications implemented do not detrimentally affect an individual’s inherent facial attributes [1]. Orthodontic therapy aims to rectify dental discrepancies and enhance aesthetics, making the treatment planning process inherently complex. In the course of rectifying occlusal or bite-related issues, there may be a decline in facial appeal. Such repercussions may arise from a misapprehension of aesthetic objectives or an insufficient regard for facial aesthetics throughout the treatment process. The attractiveness of the face is predominantly determined by its skeletal framework, which encompasses critical characteristics such as nasal structure, chin projection, and bilateral malar prominences [2].

The prominence of the malar region plays a crucial role in shaping the lateral contour of facial structures and significantly influences facial aesthetics. Malar prominence, the zygomatic or malar process of the maxilla, is characterized by a triangular morphology situated at the convergence of the anterior, zygomatic, and orbital surfaces [3]. This anatomical structure delineates a subtle contour between the lower eyelid and zygomatic bone. For a comprehensive assessment of malar eminence, a tangential line aligned with the anterior surface of the cornea has been suggested. Ideally, the projection of the malar eminence should extend approximately 2 mm beyond this line [3,4].

While the midfacial region exerts a considerable impact on facial aesthetics, there is a conspicuous deficiency of standardized diagnostic parameters within the orthodontic literature on midfacial deficiencies. To evaluate and quantify aesthetic facial profiles, a variety of analyses of both hard and soft tissues have been developed concurrently with the advancement of cephalometric methodologies [4]. Besides facilitating diagnosis and treatment planning, lateral cephalograms are instrumental in forecasting hard and soft tissue responses to orthodontic interventions. This is attributable to their capacity for straightforward acquisition, quantification, and comparative analysis of hard tissue structures via superimposition techniques. Arnett's facial soft tissue analysis is acknowledged in the scholarly literature for its thorough assessment of frontal and sagittal soft tissue dimensions [3,4]. Nonetheless, a notable constraint of this analysis is its tailored design, specifically for surgical cases [5].

The sella-nasion-orbitale (SNO) angle represents the latest development in cephalometry for evaluating malar projections using cephalometric landmarks in conjunction with lateral facial photographs of an individual [6]. Doddamani et al. [7] conducted a study to compare visual and cephalometric methods using the SNO angle to assess malar prominence and found significant skeletal disparities between the positive and negative vector cohorts, as determined by SNO angles. The SNO angulations within the negative vector cohort were, on average, 5.9° less than those in the positive vector cohort. Established positive and negative vector relationships were thoroughly documented using this methodological approach. Nevertheless, the cephalometric points employed in this investigation exhibit variability and are subject to modification due to age and bony remodeling within an individual. Consequently, there is a pressing need to implement stable landmarks.

Certain cephalometric landmarks exhibit stability throughout adulthood and are not subject to ongoing osseous remodeling; among these is the key ridge point, designated as the K point [8]. The term "key ridge" denotes a robust bony formation that emanates anteriorly from the zygomatic bone to the maxilla, supporting the maxillary first molar. In the adult population, this ridge is superior to the mesiobuccal root of the maxillary first molar. A maxillary radiograph provides a definitive visualization of the dentition's alignment relative to the key ridge. This vantage point facilitates a comprehensive assessment of the accurate arrangement of teeth within the cranial structure and yields valuable information regarding arch configuration concerning adjacent bony elements. Furthermore, the posterior palatine suture is frequently discernible from this radiographic perspective, acting as an additional reference point to enhance precision [9].

The "walker point" and the "wing point," collectively called the "double W" plane or the "DW" plane, constitute a significant component in craniofacial analysis. The DW plane is a methodologically sound approach for accurately delineating the skeletal interrelationship between the mandible and the maxilla. It systematically evaluates variations in linear dimensions to establish the sagittal jaw relationship, quantifies linear metrics to gauge the vertical height of the maxilla, and incorporates angular measurements to analyze the rotational dynamics of the jaw [10].

The presence of maxillary hypoplasia, as characterized by deficient malar development, results in what is referred to as a negative vector relationship, whereas maxillary prognathism, as characterized by malar prominence, is referred to as a positive vector [5]. Consequently, this study aimed to present a novel perspective for evaluating malar prominence, referred to as the double W-key ridge (DWK) angle (the angle formed between the DW plane and the K point), and to evaluate the anteroposterior positioning of malar prominence in young adolescents with positive and negative vector profiles using SNO and DWK angle measurements obtained from lateral cephalograms. The objective of this study was to compare the mean SNO and DWK angles between positive and negative vector profile groups, assess the correlation between SNO and DWK angles in both vector profile groups, determine the diagnostic accuracy of SNO and DWK angles in identifying vector profiles, and examine the influence of sex on SNO and DWK angle measurements within each vector profile group. The null hypothesis posited for this study was that both angles would be used to evaluate the anteroposterior positioning of malar prominence accurately.

## Materials and methods

Study design and setting

This retrospective study was conducted on the records of patients who visited the Department of Orthodontics and Dentofacial Orthopedics, Yogita Dental College, Khed, between May 2022 and May 2023. The institutional ethical approval was waived for this study as it used the patients’ records without revealing their identity and with written consent from the patients to use their records for study purposes and publication. This study adhered to the principles of the Declaration of Helsinki. Data were anonymized and stored securely to ensure patient confidentiality.

Sample size estimation

The sample size was estimated using G Power software version 3.2.9 (Heinrich-Heine-Universität Düsseldorf, Düsseldorf, Germany). The estimated sample size of 60 individuals (30 per group) was sufficient at a power of 95% and an alpha error of 5%. The utilized effect size in the analysis was 0.87, obtained from a previous study [10], which calculated the mean difference (53.90-46.00) between the SNO angle of positive and negative vector profiles and a pooled standard deviation of 9.08.

Patients’ selection

A total of 215 records were screened, and 60 records were selected based on the eligibility criteria. Young adolescents aged 11 to 14 years with good-quality lateral cephalograms and profile photographs were chosen for this study. Patients with craniofacial anomalies, cleft lip and palate, a history of orthodontic or orthognathic treatment, a history of prior malar augmentation surgery, facial trauma, or surgery, and those with missing records were excluded from the study. Patients were categorized into two cohorts: positive and negative vector groups, with almost equal numbers of males and females. Stratified sampling was used, wherein the vector relationship within each cohort stratified the dataset. Equal numbers of positive and negative vector records were selected using simple random sampling.

Methodology

**Figure 1:**
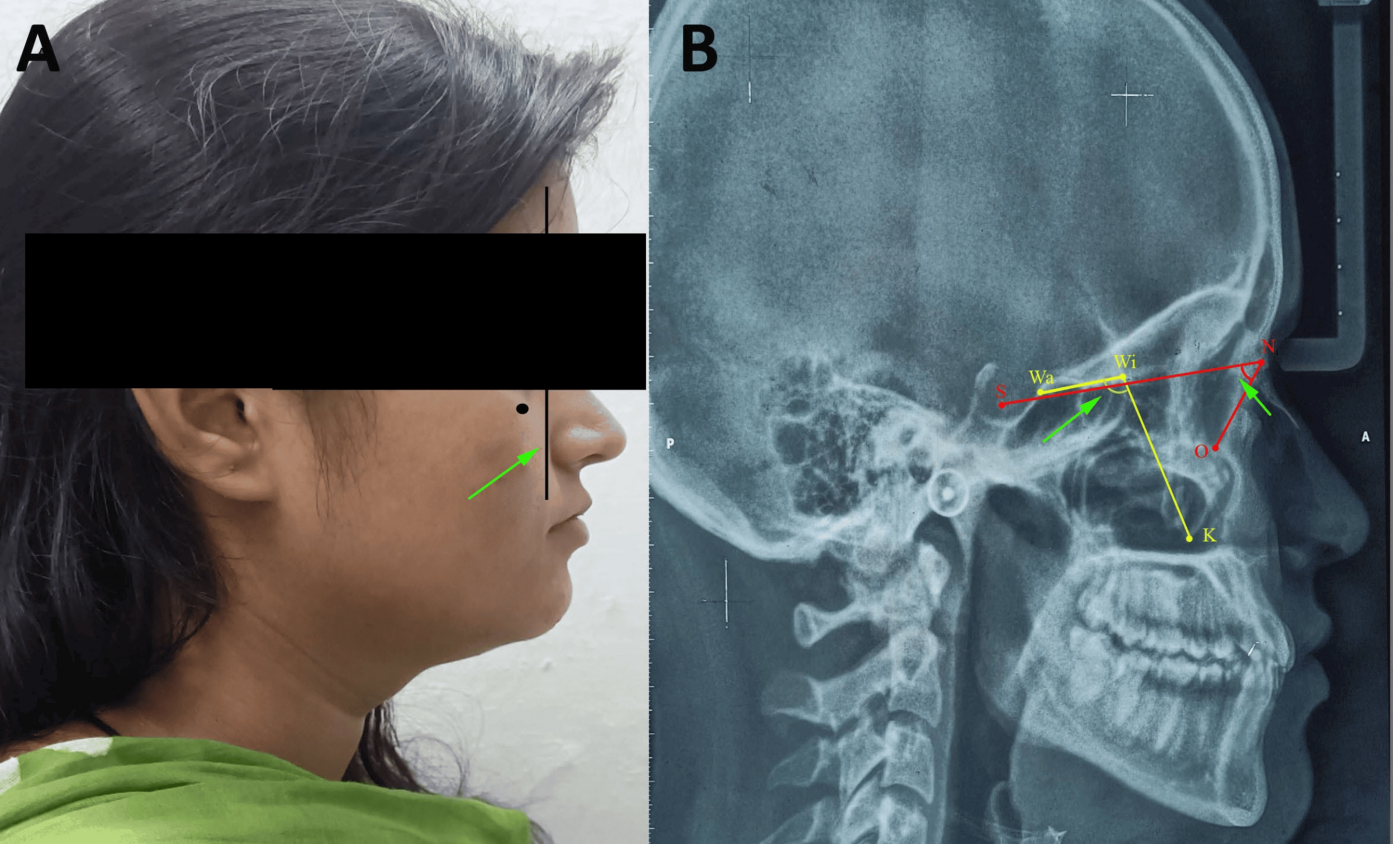
(A) Assessment of anterior malar prominence on the clinical profile photograph using vector relationship, (B) SNO angle (red) and DWK angle (yellow) SNO: sella-nasion-orbitale, DWK: double W-key ridge The image features an individual from the study and is used with her permission with written consent.

Sixty records were divided into groups based on positive and negative vectors. The positive vector group consisted of 30 individuals (13 males, 17 females) with a mean age of 12.26 ± 1.23 years, while a negative vector group included 30 individuals (16 males, 14 females) with a mean age of 11.84 ± 2.12 years. The determination of each individual’s vector relationship was based solely on profile photographs from their initial records, which were standardized by ensuring that the patient's head was oriented in the Frankfort Horizontal position. The profile photographs of the individuals were taken using a Canon 1200D camera with an 18-55 lens (Canon Inc., Tokyo, Japan), and a Godox-ML-150 macro ring flash (Godox Photo Equipment Co. Ltd., Guangdong, China) was also attached. In the current investigation, a vertical line was drawn from the apex of the cornea to the anterior malar prominence on the clinical profile photograph, serving as a valuable method for assessing malar deficiency (Figure 1A).

Lateral cephalometric images were captured using a KODAK 8000 C Digital Panoramic and Cephalometric apparatus (Carestream Health, Rochester, New York, USA) utilizing a voltage spectrum of 70 kVp and a current of 10 mA. An experienced radiologist acquired all cephalometric images. SNO angulations were used to evaluate the anteroposterior position of the malar prominence. The DW plane was marked according to walker point (the point at the intersection of the lower contours of the anterior clinoid processes and the contour of the anterior wall of the sella) and wing point (intersection of the contour of the ala major with the jugum sphenoidale). The key ridge point, or the K point, was marked, according to the study given by Weingart [8], as the lowest point on the contour of the anterior wall of the infratemporal fossa. A DWK angle between the DW plane and the K point was measured in both positive and negative vector individuals (Figure 1B).

Reliability testing

Twenty random lateral cephalograms from subjects in the study were selected, and measurements were performed twice at one-month intervals by the same observer to measure intra-observer reliability using the intraclass correlation coefficient, which yielded a value of 0.89, demonstrating excellent reliability and reproducibility. All cephalograms were traced by the same observer blinded to the group allocation.

Statistical analysis

Statistical analysis was conducted using SPSS Statistics (IBM Corp. Released 2013. IBM SPSS Statistics for Windows, Version 23.0. Armonk, NY: IBM Corp.). The normality of the data was assessed using the Shapiro-Wilk test and further confirmed through a Q-Q plot, which indicated a non-normal distribution. Continuous variables, including SNO and DWK angles, were summarized using the mean, median, standard deviation, and range. Given the non-parametric nature of the data, the Mann-Whitney U test was employed to compare the mean angles between the positive and negative vector profiles. The correlation between the SNO and DWK angles was evaluated using Spearman’s rank correlation coefficient. Sensitivity analysis for both angles was performed using the receiver operating characteristic (ROC) curve to determine their ability to identify the vector profiles. A mixed-model analysis incorporating covariates, such as group and sex, was conducted to assess their influence on the measured angles.

## Results

Descriptive analysis showed that the mean and median values of both SNO and DWK angles were higher in the positive vector group than in the negative vector group. The mean SNO angle was 53.97° (median = 54°) in the positive vector group and 44.1° (median = 44°) in the negative vector group. Similarly, the mean DWK angle was 104.2° (median = 104°) in the positive-vector group and 94.6° (median = 94°) in the negative-vector group. These findings indicate a clear distinction in angle measurements between the two vector groups (Table 1).

**Table 1:** Descriptive analysis of SNO and DWK angles in study groups Data is presented as mean and SD. SNO: sella-nasion-orbitale, DWK: double W-key ridge, CI: confidence interval, SD: standard deviation

Angle	Group	N	Mean	Median	SD	Minimum	Maximum	95% CI for mean
SNO angle in degrees	Positive vector	30	53.97	54	1.79	50	57	53.3-54.64
	Negative vector	30	44.1	44	2.23	41	48	43.27-44.93
DWK angle in degrees	Positive vector	30	104.2	104	2.06	100	108	103.43-104.97
	Negative vector	30	94.6	94	2.11	91	99	93.81-95.39

Both study groups had equal sex distributions. In the positive vector group, there were 13 (21.67%) males and 17 (28.33%) females, while the negative vector group included 16 (26.67%) males and 14 (23.33%) females. This balanced distribution ensured comparability between groups (Table 2).

**Table 2:** Distribution of sex in study groups Data is presented as n (%).

Sex	Positive vector group		Negative vector group		Total	
	n	%	n	%	n	%
Male	13	21.67%	16	26.67%	29	48.33%
Female	17	28.33%	14	23.33%	31	51.67%
Total	30	50.00%	30	50.00%	60	100.00%

In the positive vector group, the mean SNO angle was 54.31° (median = 54°) for males and 53.71° (median = 54°) for females. In the negative vector group, males had a mean SNO angle of 44.13° (median = 44.5°), while females had a mean angle of 44.07° (median = 43.5°). For the DWK angle, the mean value in the positive vector group was 104.23° (median = 104°) for males and 104.18° (median = 104°) for females. In the negative vector group, males had a mean DWK angle of 94.31° (median = 94°), while females had a mean of 94.93° (median = 94.5°). These findings indicate that while SNO and DWK angles were distinctly different between the positive and negative vector groups, the values remained relatively consistent between males and females within each group (Table 3).

**Table 3:** Descriptive analysis of SNO and DWK angles in study groups and sex Data is presented as mean and SD. SNO: sella-nasion-orbitale, DWK: double W-key ridge, CI: confidence interval, SD: standard deviation

Angle	Group	Sex	Mean	Median	SD	Minimum	Maximum	95% CI for mean
SNO angle in degrees	Positive vector	Males	54.31	54	1.80	51	57	53.22-55.39
		Females	53.71	54	1.79	50	56	52.78-54.63
	Negative vector	Males	44.13	44.5	2.22	41	48	42.94-45.31
		Females	44.07	43.5	2.34	41	48	42.72-45.42
DWK angle in degrees	Positive vector	Males	104.23	104	2.05	101	107	102.99-105.47
		Females	104.18	104	2.13	100	108	103.08-105.27
	Negative vector	Males	94.31	94	2.24	91	99	93.12-95.51
		Females	94.93	94.5	1.98	92	99	93.79-96.07

The null hypothesis was accepted for this study as both angles could be used to assess the position of malar prominence. Spearman rank correlation analysis demonstrated a strong statistically significant positive correlation (r=0.74, p=0.001) between the SNO and DWK angles. This indicated a strong association between the two measurements, suggesting that as the SNO angle increased, the DWK angle also tended to increase. The mixed model analysis demonstrated a statistically significant effect of SNO and DWK angles (p=0.001) and group (p=0.001), indicating that the angle measurements significantly differed between vector groups. However, the interaction between the repeated-measures factor and group was not statistically significant (p=0.737), suggesting no interaction effect (Table 4).

**Table 4:** Mixed model analysis for SNO and DWK angle with group as covariate *p-value < 0.05: significant SNO: sella-nasion-orbitale, DWK: double W-key ridge, df: degree of freedom, RM: repeated measure

Parameters	Sum of squares	df	Mean square	F-value	p-value
SNO angle, DWK angle	76104.03	1	76104.03	16261.94	0.001*
Group	2842.13	1	2842.13	755.36	0.001*
RM factor x group	0.53	1	0.53	0.11	0.737

The mixed-model analysis showed a statistically significant effect of SNO and DWK angles (p=0.001), confirming their relevance in this study. However, sex (p=0.473) and the interaction between the repeated measures factor and sex (p=0.467) were not statistically significant, indicating that sex did not significantly influence angle measurements (Table 5).

**Table 5:** Mixed-model analysis for SNO and DWK angle with sex as covariate *p-value < 0.05: significant SNO: sella-nasion-orbitale, DWK: double W-key ridge, df: degree of freedom, RM: repeated measure

Parameters	Sum of squares	df	Mean square	F-value	p-value
SNO angle, DWK angle	76104.03	1	76104.03	16379.86	0.001*
Sex	27.23	1	27.23	0.52	0.473
RM factor x sex	2.49	1	2.49	0.54	0.467

The Mann-Whitney U test revealed a statistically significant difference in the mean ranks of the SNO and DWK angles between the positive and negative vector groups. The strong correlation (r=0.86) suggests a high degree of association between angle measurements and vector classification (Table 6).

**Table 6:** Comparison of mean value of SNO and DWK angle with Mann-Whitney U test *r-value > 0.70: a very strong relationship, **p-value < 0.05: significant SNO: sella-nasion-orbitale, DWK: double W-key ridge

Angle	Group	n	Mean rank	Sum of ranks	p-value	r-value
SNO angle in degrees	Positive vector	30	45.50	1365	0.001**	0.86*
	Negative vector	30	15.50	465		
DWK angle in degrees	Positive vector	30	45.48	1364.5	0.001**	0.86*
	Negative vector	30	15.52	465.5		

**Figure 2:**
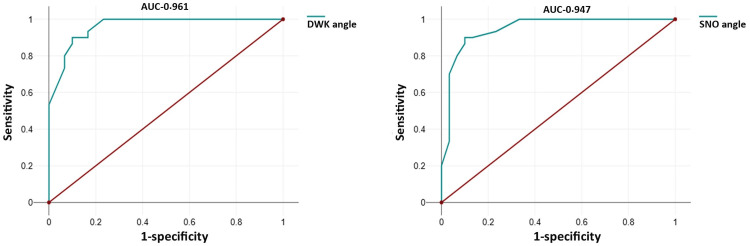
ROC curve for SNO and DWK angle AUC: area under the curve (0.9-1: accuracy is excellent), SNO: sella-nasion-orbitale, DWK: double W-key ridge, ROC: receiver operating characteristic The image is the author's original work created using data obtained from the study.

Sensitivity analysis revealed that both the SNO and DWK angles demonstrated excellent diagnostic accuracy in identifying vector profiles. The area under the curve (AUC) values were 0.947 for SNO and 0.961 for DWK, indicating a strong predictive ability (Figure 2).

The sensitivity, specificity, and precision were identical for both angles at 90%, suggesting a comparable reliability (Table 7).

**Table 7:** Sensitivity analysis of SNO and DWK angle AUC: area under the curve, SNO: sella-nasion-orbitale, DWK: double W-key ridge

Performance metrics	SNO angle	DWK angle
AUC	0.947	0.961
Sensitivity	0.900	0.900
Specificity	0.900	0.900
Precision	0.900	0.900

## Discussion

The current investigation sought to assess the anteroposterior position of zygomatic prominence in young adolescents exhibiting both positive and negative vector profiles using SNO and DWK angle measurements derived from lateral cephalometric radiographs. These results yield substantial insights into the classification of vector profiles through cephalometric evaluation, which may possess clinical ramifications in the fields of orthodontics, maxillofacial surgery, and aesthetic treatment strategies.

The research findings indicated a notable disparity in the SNO and DWK angles when comparing positive and negative vector profiles. The average SNO angle was elevated in the positive vector cohort (53.97°) in contrast to the negative vector cohort (44.1°), suggesting that subjects with a positive vector profile are inclined to exhibit a more anterior displacement of the malar prominence. Likewise, the average DWK angle was higher in the positive vector cohort (104.2°) than in the negative vector cohort (94.6°), thereby reinforcing the unique skeletal attributes that differentiate these two cohorts. These results imply that cephalometric metrics, such as SNO and DWK angles, may be utilized as dependable parameters for the identification and classification of vector profiles. Similar findings have been reported by Frey [6] and Basak et al. [11]. The positive and negative vector associations may prove beneficial in the categorization of anterior malar support during both micro- and macroaesthetic assessments of patients [12].

In accordance with the principles of growth and development, growth induces a secondary displacement of the anterior malar complex in a downward and forward trajectory, facilitated by the deposition of new bone in an upward and backward orientation. Additionally, there is a resorption process affecting the anterior maxilla and zygoma. Remodeling of the supraorbital rim and the lateral nasal complex is critical for anterior growth [13]. Furthermore, the deposition of new bone in the midfacial region transpires on the lateral zygoma and zygomatic arch, thereby contributing to an enhancement of the lateral malar prominence and maintenance of facial width in alignment with the jaws. This bone deposition within the midfacial area results in relative bulging of the nose, supraorbital rim, and potentially the lateral malar complex. Conversely, the anterior section of the malar complex displays comparatively diminished prominence, thereby enabling clinicians to assess and determine malar retrusion or protrusion during the initial phases of development [14,15]. Deficient malar development is usually associated with maxillary jaw retrusion, as seen in Class III cases, and therefore requires forward movement of the maxilla by reverse pull headgear in growing age and Le Fort 1 osteotomy with maxillary advancement in adults [15,16].

The investigation utilized sensitivity analysis, which demonstrated considerable diagnostic precision for both angles. These findings indicate that both angles possess substantial predictive capability for distinguishing between positive and negative vector profiles, thus rendering them significant instruments for clinical evaluation. Consequently, both clinical and cephalometric parameters can be employed to evaluate malar prominence in patients, thereby facilitating the planning of cosmetic surgical interventions, such as malar augmentation. As no previous study has performed a sensitivity analysis, this finding could not be supported by previous studies.

The DWK angle uses stable landmarks, such as the walker point, wing point, and key ridge point. The maturation of the middle cranial base occurs at an earlier stage, which is attributable to its role in safeguarding the brain and other essential organs. Consequently, the established stability of the middle cranial base after age eight years renders it an exemplary reference point for examining facial growth. It has been well-documented that the tuberculum sella (T) and wing point (W) landmarks, situated at the middle cranial base, exhibit a marked degree of stability [17,18]. The SNO angle utilizes the nasion point (N) and sella tunica point (S), where the consistency of the N point, as well as that of the S point, has been extensively analyzed, revealing that these landmarks demonstrate considerable variability throughout the growth process [19,20]. Moreover, the Or point has been reported as the least reliable point for cephalometric tracing [21]. Therefore, the DWK angle is more reliable than the SNO angle and can be used at any age.

Statistical evaluations additionally demonstrated that sex did not exert a significant effect on the SNO and DWK angle metrics within each vector profile category. The mixed-model analysis suggested that although the angles themselves were significant in distinguishing the vector profiles, gender did not have a statistically significant influence. This outcome is pivotal because it indicates that vector profiling based on SNO and DWK angles can be employed universally among both male and female adolescents without necessitating sex-specific modifications. The lack of a significant interaction effect between repeated measures and group categorization further suggests that the variability in measurements remains consistent across different vector profiles. Similar findings have been reported by Basak et al. [11].

Strengths of the study

One of the notable advantages of this investigation is its rigorous methodological framework, which encompasses a clearly articulated sample selection procedure and implementation of standardized imaging modalities. The study utilized stringent inclusion parameters, focusing solely on young adolescents between the ages of 11 and 14 years who possessed high-quality lateral cephalometric radiographs and standardized profile images. By systematically excluding participants with craniofacial deformities, prior orthodontic or orthognathic interventions, and those with a documented history of facial trauma or surgical procedures, the study achieved a uniform sample, thus mitigating potential confounding factors. In addition, the assessment of intra-observer reliability revealed exceptional reproducibility, signifying a substantial degree of consistency in the measurements and diminishing the potential for observer bias.

Clinical implications of the study

Differentiating between positive and negative vector profiles using cephalometric angles can aid orthodontists, maxillofacial surgeons, and aesthetic practitioners in treatment planning. For instance, individuals with a negative vector profile may benefit from orthodontic interventions or surgical procedures to enhance midfacial projections, such as malar augmentation or orthognathic surgery. Additionally, these measurements can be used in preoperative assessments to predict surgical outcomes and in postoperative evaluations to monitor changes in midfacial morphology.

Limitations and future recommendations

First, the study's retrospective design constrained its capacity to ascertain causal relationships. Although the research delineated significant disparities among vector profiles, it lacks insight into the developmental determinants contributing to these discrepancies. A longitudinal study framework would prove more efficacious in monitoring alterations in SNO and DWK angles over time and comprehending their ramifications in facial growth and development. Second, while the investigation concentrated on adolescents, the results may not be readily generalizable to other demographic cohorts, particularly adults, who may demonstrate distinct skeletal characteristics attributable to aging and soft-tissue modifications. Another constraint is the dependence on two-dimensional cephalometric imaging, which, although commonly utilized, has inherent limitations in evaluating three-dimensional facial configurations. Future investigations could integrate three-dimensional imaging modalities such as cone-beam computed tomography to facilitate a more exhaustive assessment of malar prominence and vector profiling. Moreover, the study did not incorporate variations in soft tissues, which are pivotal in determining overall facial aesthetics. Integrating soft tissue analysis with skeletal metrics would yield a more comprehensive methodology for vector profiling.

## Conclusions

This study confirmed that the DWK angle is a reliable and stable cephalometric parameter for differentiating between adolescents' positive and negative vector profiles. DWK was smaller in the negative vector group than in the positive vector group, with no sexual dimorphism. The findings demonstrate a significant strong correlation between SNO and DWK angles and the high diagnostic accuracy of both angles in assessing malar prominence. The results suggest that these measurements can be effectively utilized in orthodontics, maxillofacial surgery, and aesthetic treatment planning, offering a valuable tool for clinical decision-making and patient evaluation.
